# Technical Note: Comparison of the internal target volume (ITV) contours and dose calculations on 4DCT, average CBCT, and 4DCBCT imaging for lung stereotactic body radiation therapy (SBRT)

**DOI:** 10.1002/acm2.13041

**Published:** 2020-10-12

**Authors:** Michael Dumas, Eric Laugeman, Parag Sevak, Karen C. Snyder, Weihua Mao, Indrin J. Chetty, Munther Ajlouni, Ning Wen

**Affiliations:** ^1^ Department of Radiation Oncology Henry Ford Health System Detroit MI USA; ^2^ Department of Radiation Oncology Washington University St. Louis MO USA; ^3^ Department of Radiation Oncology Columbus Regional Health Columbus IN USA

**Keywords:** 4DCBCT, 4DCT, lung SBRT, respiratory motion

## Abstract

**Purpose:**

To investigate the differences between internal target volumes (ITVs) contoured on the simulation 4DCT and daily 4DCBCT images for lung cancer patients treated with stereotactic body radiotherapy (SBRT) and determine the dose delivered on 4D planning technique.

**Methods:**

For nine patients, 4DCBCTs were acquired before each fraction to assess tumor motion. An ITV was contoured on each phase of the 4DCBCT and a union of the 10 ITVs was used to create a composite ITV. Another ITV was drawn on the average 3DCBCT (avgCBCT) to compare with current clinical practice. The Dice coefficient, Hausdorff distance, and center of mass (COM) were averaged over four fractions to compare the ITVs contoured on the 4DCT, avgCBCT, and 4DCBCT for each patient. Planning was done on the average CT, and using the online registration, plans were calculated on each phase of the 4DCBCT and on the avgCBCT. Plan dose calculations were tested by measuring ion chamber dose in the CIRS lung phantom.

**Results:**

The Dice coefficients were similar for all three comparisons: avgCBCT‐to‐4DCBCT (0.7 ± 0.1), 4DCT‐to‐avgCBCT (0.7 ± 0.1), and 4DCT‐to‐4DCBCT (0.7 ± 0.1); while the mean COM differences were also comparable (2.6 ± 2.2mm, 2.3 ± 1.4mm, and 3.1 ± 1.1mm, respectively). The Hausdorff distances for the comparisons with 4DCBCT (8.2 ± 2.9mm and 8.1 ± 3.2mm) were larger than the comparison without (6.5 ± 2.5mm). The differences in ITV D95% between the treatment plan and avgCBCT calculations were 4.3 ± 3.0% and −0.5 ± 4.6%, between treatment plan and 4DCBCT plans, respectively, while the ITV V100% coverages were 99.0 ± 1.9% and 93.1 ± 8.0% for avgCBCT and 4DCBCT, respectively.

**Conclusion:**

There is great potential for 4DCBCT to evaluate the extent of tumor motion before treatment, but image quality challenges the clinician to consistently delineate lung target volumes.

## 1 INTRODUCTION

For early‐stage lung cancer patients, stereotactic body radiotherapy (SBRT) has become one of the primary treatment options.^1^ Improvements in patient localization using on‐board imaging and cone‐beam computed tomography (CBCT) and the ability to account for tumor motion have led to the increase in lung SBRT treatments.^2^ One strategy for tumor motion management is the use of four‐dimensional CT (4DCT) simulations to determine the total extent of motion for planning purposes.^3^, ^4^ Then a 3D CBCT is used for online patient setup and the patient is treated free breathing. This strategy, however, does not take into account potential variations in a patient’s breathing pattern from simulation to treatment.

The four‐dimensional CBCT is now becoming a clinically feasible tool in the treatment room.^5^, ^6^ The advantage is the ability to visualize the motion of the tumor at the time of treatment. While a phantom study suggested image guidance with 3DCBCT has similar accuracy to 4DCBCT image guidance,^7^ a clinical study showed improvement in target localization with 4DCBCT as tumor motion increased.^6^ Several investigators have looked at 3DCBCT for dose accumulation and adaptive planning of SBRT lung treatment.^8^, ^9^ Even so, the issue of calculating dose on a moving target exists.^10^ Similar to the advent of 4DCT, 4DCBCT can be employed to determine dose delivered to a moving tumor at the time of treatment.

This study evaluated the internal target volume (ITV) of clinically treated patients by comparing the contours on 10 phases of the 4DCBCT with the treatment planning ITV based on the 4DCT. Dose to the moving tumor was calculated on each phase of the 4DCBCT, and ITV coverage was compared to the treatment plan. Eclipse 4DCBCT dose calculations were checked with ion chamber measurements in a CIRS anthropomorphic lung phantom.

## 2 METHODS

Nine patients with non‐small‐cell lung cancer underwent a 4DCT with a Philips Big Bore Brilliance CT Simulator (Philips Healthcare, Massachusetts, USA). Patients were immobilized in an Elekta BodyFix BlueBAG (Stockholm, Sweden). The Philips Bellows Belt was used to capture the breathing trace. Projections were retrospectively binned and reconstructed into four phases based on the phase breathing trace. Clinically, four phases are utilized for planning. Average CT (avgCT) and maximum intensity projection (MIP) datasets were generated from the phases. The ITV was contoured on the 4DCT MIP (4DCT ITV), and evaluated on each breathing phase. A 3‐mm isotropic margin was placed around the ITV to create the planning target volume (PTV). Treatment plans were generated for a Varian EDGE linear accelerator (Varian, Palo Alto, CA) with a 6 MV flattening filter free beam. Treatment plans were prepared on the avgCT using Eclipse’s AAA algorithm version 11, with prescription doses of 40‐56 Gy in four fractions. Planning criteria was based on RTOG 0915,^11^ such that at least 95% of the PTV is covered by the prescription dose. Key plan metrics evaluated include V100% (percent of target covered by the prescription dose) and D95% (dose covering 95% of the target).

**Fig. 1:**
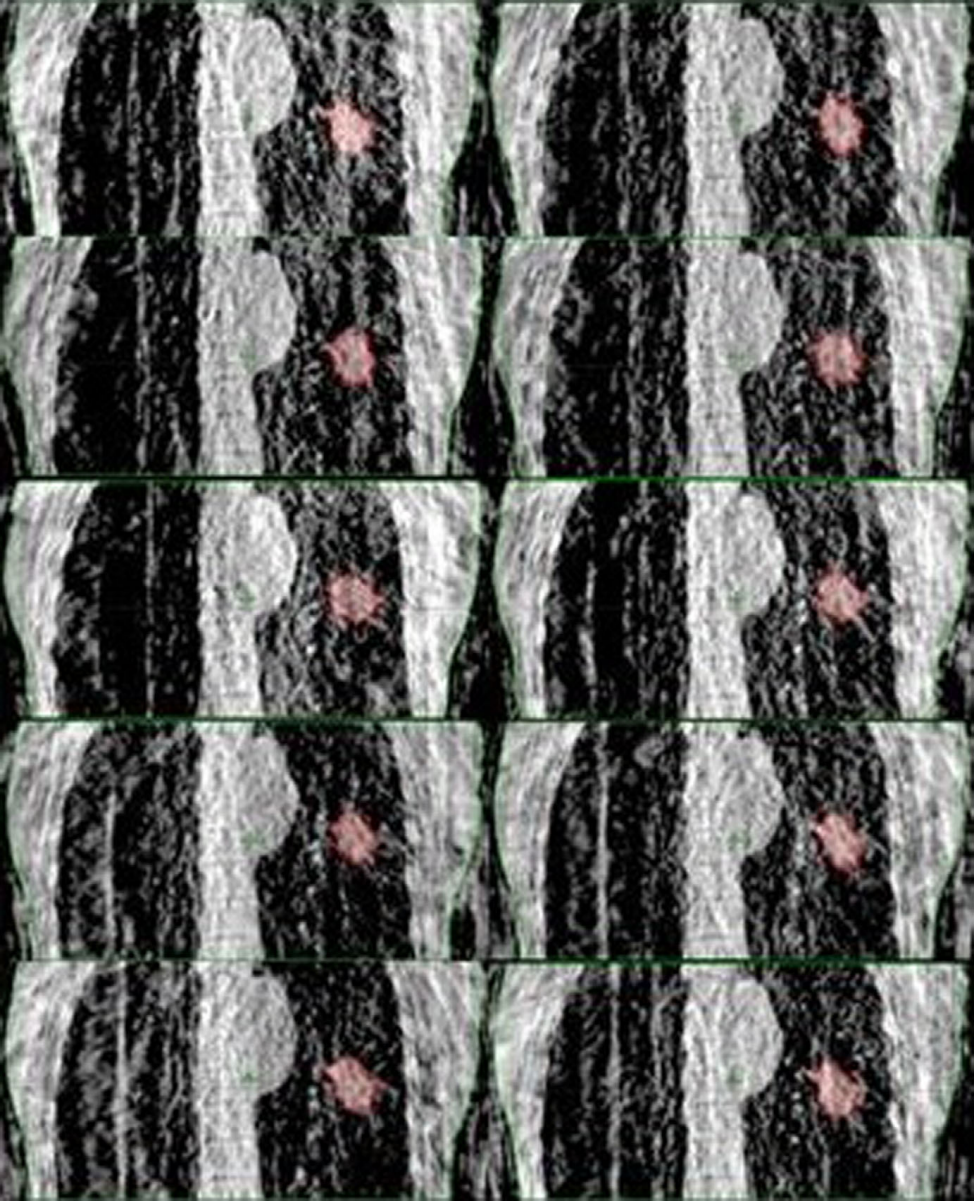
Four dimensional CBCT ITV. Tumor volumes contoured on 10 phases (red). The union of the 10 phases comprised the 4DCBCT ITV.

For each fraction, a free‐breathing 4DCBCT was acquired. Varian Real‐time Position Management (RPM) was used to track patient breathing. The RPM block was placed on the patient between the xiphoid and umbilicus for diaphragmatic displacement tracking. A total of 1800 projections were acquired over a full gantry rotation at a speed of 3 degrees per second. For patient localization a 3DCBCT (avgCBCT) was reconstructed (filtered back‐projection) online using every projection. The same projections used for localization were binned into 10 phases based on the RPM breathing trace and reconstructed offline. A radiation oncologist contoured the target volume on the avgCBCT (avgCBCT ITV) and each 4DCBCT phase (Fig. 1).

The 4DCBCT ITV was formed by the union of the 10 contours. The Dice coefficient, Hausdorff distance, and center of mass difference between the 4DCT ITV, avgCBCT ITV, and 4DCBCT ITV were computed with in‐house software. The Dice coefficient quantifies the overlap between two contours by the ratio of twice the intersection divided by the sum of the contour volumes. The Hausdorff distance is the greatest minimum distance between all vertices of the two contours. Mean values for each patient (over four fractions) as well as mean of the means over nine patients (group systematic error) and standard deviation of means (SD of the systematic error) were calculated.

Using the online registration, the treatment plans were calculated on each phase of the 4DCBCT and the avgCBCT for each fraction of nine patients. The average treatment metrics over four fractions, D95%, and V100%, were compared with the coverages achieved in the original treatment plans. It was assumed that the length of each of the 10 phases was the same, thus each treatment field contributed 1/10 of its MU to each phase. A plan sum of all the phases yielded the single fraction dose delivered to the target.

**Fig. 2:**
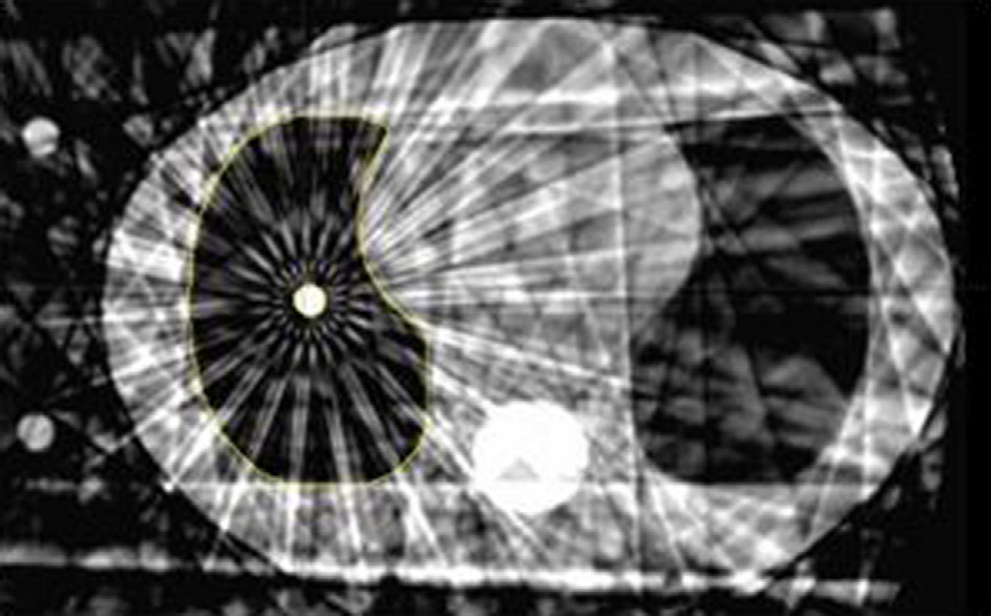
A single 4DCBCT phase of the CIRS lung phantom used for dose measurements. Note the streaking artifacts and overall noisiness of the image.

Patient‐specific calculations and measurements were acquired for each plan using a CIRS lung phantom. A 10‐phase 4DCBCT of the phantom was acquired (Fig. 2), and patient plans were mapped to and calculate on each phase of the phantom’s scan. The interphase average coefficient of variation (CV) was determined using all 76 treatment fields to quantify how uncertainty created from CBCT imaging noise affects the dose calculation accuracy. The interphase average CV was found by computing the standard deviation in calculated dose over the 10 phases of the 4DCBCT in each treatment field and averaging all treatment fields together. Point dose measurements were acquired with an IBA CC01 ion chamber at isocenter to test the calculation algorithm’s precision for 4DCBCT based dose calculations. The ion chamber was calibrated in solid water against the treatment‐planning system the day of the plan measurements.

## 3 RESULTS

### 3.1 Contouring

Table 1 shows the three contour comparisons performed over the four treatment fractions: 4DCT ITV against avgCBCT ITV, 4DCT ITV against 4DCBCT ITV, and avgCBCT ITV against 4DCBCT ITV. The 4DCT ITV against avgCBCT ITV comparison (section 1) evaluates the image quality and contour differences between simulation and online setup. The 4DCT ITV against 4DCBCT ITV comparison (section 2) examines differences resulting from slice versus planar acquisition methods and slower gantry acquisition speed of the 4DCBCT. The avgCBCT ITV against 4DCBCT ITV comparison (section 3) outlines the contour differences in the 3D acquisition used for online positioning and the 4D acquisition to assess the tumor motion envelope. Dice coefficients were equivalent between each comparison (0.7 ± 0.1 with p = 0.8). Similarly, the center of mass differences were consistent between the comparisons: 2.3 ± 1.2, 3.1 ± 1.6, and 2.6 ± 2.1 mm, respectively (p = 0.6). The Hausdorff distances showed no statistical differences (p = 0.4), however comparisons in section 2 and 3 (8.2 ± 2.9 mm and 8.1 ± 3.2 mm) were larger than section 1 (6.5 ± 2.5 mm).

**Table 1:** Comparison of 4DCT, average CBCT, and 4DCBCT ITV contours using the following evaluation metrics: Hausdorff distance, center of mass difference, and dice coefficient.

Patient	4DCT ‐ Average CBCT (1)						4DCT ‐ 4DCBCT (2)						Average CBCT ‐ 4DCBCT (3)					
	Hausdorff distance (mm)		COM (mm)		dice coefficient		Hausdorff distance (mm)		COM (mm)		dice coefficient		Hausdorff distance (mm)		COM (mm)		dice coefficient	
	mean	SD	mean	SD	mean	SD	mean	SD	mean	SD	mean	SD	mean	SD	mean	SD	mean	SD
1	3.5	0.4	1.3	0.3	0.8	0.0	5.0	0.9	1.5	0.7	0.8	0.0	4.9	0.7	1.2	0.3	0.9	0.1
2	5.1	0.5	0.9	0.4	0.8	0.0	6.0	0.7	1.8	0.6	0.9	0.0	7.2	0.5	1.5	0.6	0.8	0.0
3	5.3	0.5	1.5	0.7	0.8	0.0	6.8	0.5	1.7	0.8	0.8	0.0	7.0	0.6	1.5	0.4	0.8	0.0
4	3.8	0.8	1.2	0.6	0.8	0.0	7.0	1.0	2.4	0.4	0.6	0.1	7.3	1.1	1.9	0.9	0.6	0.1
5	3.7	0.8	1.8	0.5	0.8	0.1	4.3	0.3	1.9	0.5	0.8	0.0	3.9	1.0	0.7	0.3	0.9	0.1
6	8.6	0.9	2.5	1.6	0.6	0.1	11.6	1.2	3.2	0.4	0.4	0.1	7.7	0.8	2.3	0.8	0.8	0.1
7	9.9	1.2	2.3	0.6	0.6	0.0	11.8	1.1	6.2	1.1	0.7	0.0	13.3	1.3	7.0	1.1	0.5	0.1
8	9.2	1.1	4.3	3.0	0.6	0.0	9.0	1.7	5.0	2.7	0.7	0.0	7.2	2.0	1.4	0.6	0.7	0.1
9	9.1	0.7	4.5	1.5	0.8	0.0	12.5	1.3	4.2	2.2	0.6	0.1	14.1	2.8	6.1	0.8	0.6	0.1
Mean Group Error (M)	6.5		2.3		0.7		8.2		3.1		0.7		8.1		2.6		0.7	
Systematic Error (Σ)	2.5		1.2		0.1		2.9		1.6		0.1		3.2		2.1		0.1	
Random Error (σ)	0.8		1.3		0.0		1.0		1.3		0.0		1.4		0.7		0.1	

### 3.2 Dose evaluation

Table 2 tabulates the dose coverage comparisons over the four fractions of the treatment. The overall prescription dose coverage (V100%) of the avgCBCT ITV and 4DCBCT ITV were 99.0 ± 1.9% and 93.1 ± 8.0%. For tumor motion ≥1 cm the coverage dropped to 98.3 ± 1.0% and 88.1 ± 3.8%, respectively. The aggregate difference in D95% between the treatment plan versus avgCBCT and treatment plan versus 4CBCT were 4.3 ± 3.0% and −0.5 ± 4.6%, respectively. For tumors with < 1 cm of motion, ΔD95% increased 1.4% for 4DCBCT. While tumors with ≥1 cm of motion had the ΔD95% decrease by approximately 2.6%. Tumor motion did not affect ΔD95% for the avgCBCT.

**Table 2:** Calculated prescription dose coverage (V100%) and dose to 95% (D95%) of the ITV for avgCBCT and 4DCBCT scans.

Tumor motion	Patient	Avg CBCT		4DCBCT		Difference (4D‐3D)	
		V100% (%)	D95% (Gy)	V 100% (%)	D95% (Gy)	V 100% (%)	D95% (%)
<1 cm motion	1	100 ± 0	3.3 ± 0.1	99 ± 0	2.6 ± 0.2	−1 ± 0	‐1.4 ± 0.2
	2	100 ± 0	3.3 ± 0.1	100 ± 0	4.0 ± 0.0	0 ± 0	1.3 ± 0.1
	3	100 ± 0	6.5 ± 0.1	99 ± 1	4.3 ± 0.2	−1 ± 1	‐4.7 ± 0.3
	4	100 ± 0	7.9 ± 0.1	93 ± 4	−2.5 ± 0.9	−7 ± 2	‐21 ± 0.9
	5	100 ± 0	4.8 ± 0.2	100 ± 0	3.0 ± 0.2	0 ± 0	‐3.7 ± 0.3
	6	96 ± 3	−0.4 ± 0.3	83 ± 6	−5.8 ± 0.7	−13 ± 3	‐11 ± 0.8
>1 cm motion	7	100 ± 0	5.2 ± 0.2	91 ± 3	−3.5 ± 0.7	−9 ± 2	‐18 ± 0.8
	8	100 ± 0	8.1 ± 0.1	97 ± 3	3.1 ± 0.9	−3 ± 1	‐10 ± 0.9
	9	95 ± 3	−0.4 ±.5	76 ± 6	−9.3 ± 1.0	−19 ± 3	‐18 ± 1.1
Overall Average		99 ± 2	4.3 ± 3.0	93 ± 8	−0.5 ± 4.6	−6 ± 6	−9.8 ± 7.6
Average with < 1cm of motion		99 ± 1	4.2 ± 2.6	96 ± 2	0.9 ± 3.7	−4 ± 1	−6.8 ± 7.4
Average with> 1cm of motion		98 ± 1	4.3 ± 3.5	88 ± 4	−3.1 ± 5.0	−10 ± 3	−15.3 ± 3.8

Average and standard deviation values were calculated over the four‐fraction treatment course. The largest differences were found in patients 6 and 9. This discrepancy is due to the poor image quality in the AvgCBCT and 4DCBCT, caused by pleural effusion and poor chest wall‐tumor contrast.

The overall interphase CV was 3.8 ± 1.4% over 10 phases. Point dose measurements per patient plan showed excellent agreement with 4DCBCT dose calculations with an average dose difference of 0.1 ± 1.0%. Over all 76 treatment beams the average dose difference was 0.1 ± 2.7%. Per field calculation versus measured dose differences did not exceeded 10% for any patient. These measurements provide a basis for 4DCBCT dose calculations.

## 4 DISCUSSION

This study sought to quantify the capability to contour on 4DCBCTs and determine the dose delivered to patients receiving lung SBRT. The 4DCBCT image quality affected the physician’s ability to identify the tumor volume. Streak artifacts, as well as lesions adjacent to high intensity anatomy (blood vessels, the diaphragm, and the chest wall), reduced tumor contrast. Additionally, tumor size and composition may change over the course of treatment.^12^

Contrast to noise ratio for CBCT has been shown to decrease linearly with faster gantry speed and fewer projections.^13^ For this study a compromise was made between scan time and number of projections per phase on the 4DCBCT. A scan speed of 3°/second was selected to improve image quality, while doubling the typical avgCBCT scan time. Due to phase binning, 4DCBCT image quality per phase is inferior to avgCBCT.^13^ The projections are split into 10 phases, reducing the number of projections per phase approximately fivefold, relative to a standard avgCBCT using a standard CBCT speed (1 RPM).

On average the Dice coefficients had similar values across all three comparisons. The similarity of the datasets in regard to the avgCBCT ITV versus the 4DCT ITV and 4DCBCT ITV is due to the overlap in the time‐averaged motion envelope captured by the avgCBCT and the full motion envelope estimated by 4DCT and 4DCBCT.^14^, ^15^, ^16^ Similar Dice coefficients for 4DCT and 4DCBCT ITV comparisons show that although the 4D images are acquired differently (slice based vs. planar based, and daily breathing pattern variations), the ITV contours largely overlap (Dice coefficient of 0.7).

The COM differences were smallest between the 4DCT versus avgCBCT comparison (2.3 mm), which is due to the physician performing online registration with these two image sets. A larger difference was found with the avgCBCT versus 4DCBCT comparison (2.6 mm), including a systematic error of 2.1 mm. For tumor motion greater than 1 cm, the systematic shift increases to 2.4 mm. This stems from the time‐average motion envelope compared to the full motion envelope; these differences become more pronounced with greater tumor motion. This explains how the 4DCT versus 4DCBCT comparison has a higher COM difference (3.1 mm) relative to the 4DCT versus avgCBCT comparison. When the systematic shift between average and 4D acquisition methods is factored in, 4DCT versus 4DCBCT has the lowest COM difference. Therefore, online 4DCBCT patient localization would improve the targeting accuracy as tumor motion increases.

**Fig. 3:**
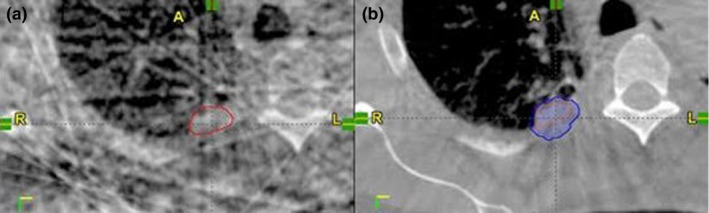
Single phase ITV (red) from patient 4 where tumor/chest wall border is indistinguishable due to significant streak artifacts in the 4DCBCT (A). Resulting 4DCBCT ITV (blue) expands posteriorly and laterally into the chest wall compared to the avgCBCT ITV (orange) and yields lower dose coverage reported to 4DCBCT ITV. This can be seen by the low Dice coefficient between avgCBCT and 4DCBCT in Table 1.

The increase in Hausdorff distances for 4DCBCT ITV results from the presence of streak artifacts that limit the ability to visualize the complete tumor motion. As seen in Fig. 3, streak artifacts decreased the discernibility of the lesion borders. In a single 4DCBCT phase, the border of the tumor and chest wall is hardly distinguishable. This led to the 4DCBCT ITV expanding posteriorly and laterally into the chest wall compared to the avgCBCT. While a large Hausdorff distance difference between avgCBCT and 4DCBCT may be expected, since the full extent of the motion is being contoured compared to an average intensity,^17^ a similar distance was also observed for the 4DCT ITV and 4DCBCT ITV comparison. This is due to the registration, image quality, and difference in breathing patterns. Another consideration is the difference between the 4‐phase 4DCT and 10‐phase 4DCBCT. Previous publications have shown that the ITV may be underestimated when contoured on two extreme phases or when the tumor motion is greater than 1.6 cm.^18^, ^19^ The work by Cao et al. demonstrated that ITVs contoured on the 4‐phase scans encompassed 94.4% of the 10‐phase ITVs.^18^

The advantage of 4DCBCT is the ability to capture the full range of tumor motion.^20^, ^21^ Shimohigashi et al^22^ showed that with increasing excursion variations on a phantom, 4DCBCT had improved target localization over avgCBCT. Sweeney et al^6^ used an Elekta Synergy to evaluate patient setup using online 4DCBCT. They showed improved localization compared to 3D with increasing tumor motion and especially with tumors adjacent to the diaphragm. Two (patients 7 and 9) patients with tumors located adjacent to the diaphragm had tumor motion ≥1 cm. These contour differences were noticeably greater, suggesting 4DCBCT localization may have improved setup accuracy. This is backed up by the Table 1 comparison of average CBCT to 4DCBT. The COM and Dice coefficients for the two patients (7,9) are much worse than the other seven patients. If the avgCBCT is used for online patient setup, an appropriately sized ITV margin should be applied to encompass the diaphragmatic tumor motion uncertainty not captured in the average motion envelope.

For Patient 6, low Dice coefficients and COM calculations between simulation 4DCT and treatment CBCTs can be explained by the development of scar tissue adjacent to the tumor. This resulted in sizably larger contours on the daily CBCTs, which did not match well with the planning CT. Compared to contouring island lesions (where the low‐density background of the lung provides high contrast), central tumors, tumors adjacent to the chest wall, and tumors surrounded by scar tissue are more challenging due to limited tumor contrast. New techniques such as iterative reconstruction may assist the clinician in contouring ITVs.^23^

The ΔV100% and ΔD95% difference between avgCBCT versus 4DCT and 4DCBCT versus 4DCT shows the magnitude of several effects. First, there is blurring of the dose resulting from a 4D dose calculation (motion blur). Secondly, the difference between the avgCBCT contour and 4DCBCT contour, which is a function of 3D versus 4D imaging as well as the image quality and physiological changes. Since tumors with less motion would exhibit minimal motion blurring, the dominant dose difference is attributed to contour variations; therefore, the contour difference alone results in drop in ΔD95% of 6%. Using this comparison as a baseline, we can see the effect of increased motion blurring and contour differences with increased motion when looking at the ΔD95% difference between avgCBCT versus CT and 4DCBCT versus CT. Two patients (6, 9) with noticeably low tumor‐chest wall contrast on the avgCBCT and 4DCBCT were removed as outliers to measure the degree that poor image quality affected the dose accumulation results. In the remaining 7‐patient set, the ΔV100% and ΔD95% 4DCBCT dose metrics markedly improve by 4%, respectively. This equates to 97.0 ± 1.6% coverage of the ITV by the prescription dose and the D95% exceeding the planned dose by 3.3 ± 6.1%. Consequently, a reasonable conjecture would be that improved contrast and image quality would allow these challenging cases to be effectively analyzed with 4DCBCT, and provide a standard consistent with easily distinguishable targets.

## 5 CONCLUSION

This study demonstrated that 4DCBCT has potential to evaluate the extent of daily tumor motion and the accumulated dose delivered to patients. These dose calculations were verified by absolute dose measurements on a lung phantom. While the calculated dose’s CV was up to 5% over 10 phase of the 4DCBCT, the measured dose agreed with calculation by 0.1 ± 1.0%.

Currently, 4DCBCT shows the greatest value for tumors with motion greater than 1 cm, although available 4DCBCT image quality can hinder the ability to contour tumor volumes. Further improvement on 4DCBCT image quality and streak artifact reduction is recommended for clinical implementation. Another way to mitigate artifacts would be to use a breath hold technique combined with fast CBCTs; this would limit organ motion, greatly reducing motion blur and streaking artifacts.^24^

## CONFLICTS OF INTEREST

None.
